# Associations among mild behavioral impairment symptoms, brain pathology and cognition in individuals with autosomal‐dominant Alzheimer’s disease: Findings from the Colombia‐Boston (COLBOS) biomarker study

**DOI:** 10.1002/alz.088002

**Published:** 2025-01-03

**Authors:** Daniel Vasquez, David Fernando Aguillon, Ana Y Baena, Stephanie Langella, Diana Munera, Victoria Zubiri, Claudia Ramos, Sergio Alvarez, Martin Ochoa‐Escudero, Elizabeth Kaplan, Celina F. Pluim McDowell, Jairo E. Martinez, Averi Giudicessi, Alex L Badillo‐Cabrera, Nikole A Bonillas Félix, Clara Vila‐Castelar, Liliana A Ramirez Gomez, Francisco Lopera, Jennifer R. Gatchel, Yakeel T. Quiroz

**Affiliations:** ^1^ Grupo de Neurociencias de Antioquia, Facultad de Medicina, Universidad de Antioquia, Medellín, Antioquia Colombia; ^2^ Grupo de Neurociencias de Antioquia, Universidad de Antioquia, Medellín Colombia; ^3^ Massachusetts General Hospital, Harvard Medical School, Boston, MA USA; ^4^ Hospital Pablo Tobón Uribe, Medellín Colombia; ^5^ Grupo de Neurociencias de Antioquia, Facultad de Medicina, Universidad de Antioquia, Medellín Colombia

## Abstract

**Background:**

Neuropsychiatric symptoms (NPS) are common in early stages of Alzheimer’s disease (AD) and may be early markers of cognitive decline and dementia in older individuals. The Mild Behavioral Impairment Checklist (MBI‐C) was developed to capture new‐onset transdiagnostic NPS in individuals at risk of dementia. We sought to determine whether mild behavioral impairment symptoms are elevated in non‐demented Presenilin‐1 (PSEN1) E280A carriers, who are genetically determined to develop dementia by their 50s. We hypothesized that those with higher MBI‐C total scores would have worse memory performance and greater brain pathology.

**Method:**

A total of 25 mutation carriers (mean age = 36.3±7.23; 56% females; 18 cognitively‐unimpaired, 7 impaired) and 30 non‐carriers (70% females) from the Colombia‐Boston (COLBOS) Biomarker study were included. All participants completed cognitive testing, the MBI‐C (self‐reported), and florbetapir (amyloid), flortaucipir (tau) PET, and structural magnetic resonance imaging. We used Mann‐Whitney Test to examine group differences, and Spearman’s correlation and linear regression to examine associations among MBI‐C total score, Geriatric Depression Scale‐15 (GDS), Mini‐Mental State Examination (MMSE), CERAD word list learning delayed recall, cortical amyloid burden, regional tau, and hippocampal volume. In sensitivity analyses, we restricted carriers to the 18 unimpaired.

**Result:**

MBI‐C total scores were similar between carriers and non‐carriers (p = 0.09). Compared to non‐carriers, carriers had greater amyloid burden (r = 0.55; p = 0.004) and lower hippocampal volume (r = ‐0.43; p = 0.034). Among all carriers, higher MBI‐C total scores were associated with lower hippocampal volume (r = ‐0.44; p = 0.034), greater amyloid burden (r = 0.5; p = 0.011), and worst memory performance (r = ‐0.36; p = 0.021). There were no associations with tau pathology (p‐values>0.05). Sensitivity analyses were consistent with previous results except for hippocampal volume (r = ‐0.4; p = 0.08).

**Conclusion:**

Findings suggest that NPS captured by MBI‐C are associated with pathology and memory in a Colombian kindred with autosomal dominant AD, years before the median age of dementia onset in this cohort. This underscores the importance of behavioral symptoms in preclinical and prodromal ADAD. Follow‐up analyses with larger samples and longitudinal assessments are needed to characterize neuropsychiatric symptom evolution as an early marker and target for intervention.

